# Early Requirement for Bioinformatics in Undergraduate Biology Curricula

**DOI:** 10.3389/fbinf.2021.656531

**Published:** 2021-04-23

**Authors:** Matthew G. Niepielko, Maria Shumskaya

**Affiliations:** ^1^ New Jersey Center for Science, Technology, and Mathematics, Kean University, Union, NJ, United States; ^2^ School of Natural Sciences, Biology, Kean University, Union, NJ, United States

**Keywords:** undergraduate education, computational biology, statistics for biologists, R, stem education

## Introduction

As the world unravels its most impactful event of the century so far – the COVID-19 pandemic, - billions of people turn on televisions, tune into radios, and browse websites trying to understand what the epidemiologic graphs are saying; and in most cases, they turn to media and friends asking to explain what these graphs mean. The COVID-19 pandemic has confirmed: there are huge gaps in the ability for the general population to interpret statistical analyses and graphical representation of biological data (Andrew, 2020; Leybzon, 2020; Tracy, 2020).

In the current situation, understanding data being a prerogative for only data specialists is gone; every health care professional, biologist, chemist, or any natural scientist have taken on the responsibility for the evaluation of massive amount of the pandemic data and delivering the conclusions to their friends and families. But are we really prepared for such work and responsibility? As classically trained in US biologists, we were never required by undergraduate college programs to dive deep into the quantitative analysis world (Cheesman et al., 2007). We were dealing with enzyme kinetics graphs in biochemistry, but not once did we touch “big data”–with exception to some of our friends who were brave enough to enroll into a biostatistics class as an elective. Only later, in graduate school, some had an opportunity to take bioinformatics courses such as computational biology, systems biology, or statistical programming.

Today, there is enough data generated by sequencing, gene expression, bench work on DNA, proteins, and metabolites, thus bioinformaticians have plenty of work to do in phylogenetics, gene expression analysis, genome analysis or an interactome prediction (Hagen, 2000; Gauthier et al., 2019). Many of bioinformaticians either have a computer science background or learned computational analysis in their graduate programs. With the overwhelming amount of data that is available today on any topic, including ecology, biodiversity, epidemiology, we believe that all biologists should receive mandatory training in bioinformatics during their undergraduate years, just as they receive training in organic chemistry or physics. For almost two decades, it has been documented that there is need for undergraduate life science majors to graduate with competency in bioinformatics to help not only scientific progression, but students’ careers as well (Bialek and Botstein, 2004; Pevzner and Shamir, 2009; Levine, 2014; American Association for the Advancement of Science, 2015; Sayres et al., 2018), with attempts to implement data science across life sciences curriculum (Dill-McFarland et al., 2021). However, there are still barriers preventing successful inclusion of bioinformatics into undergraduate life sciences education, including lack of student interest, overly full curricula, lack of student preparation, and faculty members belonging to underrepresented groups (Williams et al., 2019). Here, we discuss our opinions and experiences regarding the inclusion of bioinformatics early in undergraduate life science curricula at Kean University, a Hispanic-serving Institution (HSI).

Courses that cover bioinformatics skills should be offered as early as sophomore year, immediately after students complete two semesters of general biology courses and introductory statistics. We advocate for this program improvement because biologists need to understand basic data analysis and be familiar with the methods applied, their pros and cons. The lack of important skills necessary to evaluate the validity of a data analysis or draw a critical conclusion from a graph we observe in undergraduate biology majors is devastating. “What is p-value, why do we need it here and what does it tell us? Are graphs scaled correctly for comparison? Do the error bars represent standard error or standard deviation? Is all the data represented on the graph or is there just the averages?”–“How would I know?” These common conversations with students make it clear: the opinion of a person with a college degree in biology can be easily manipulated with some invalid data. We would not expect a biology major to excel in math; rather, we want all students to accrue basic computational analysis skills to deal with the data by embracing the Jim Frost idea: “I’ll help you intuitively understand statistics by focusing on concepts and using plain English so you can concentrate on understanding your results” (Allison Loves Math Podcast, 2021; Frost, 2021).

Possessing essential computational skills and bioinformatics tools are no longer for special people talented “in computers;” it is a required competency for a biologist (Pevzner and Shamir, 2009; White et al., 2013; Sayres et al., 2018). Computational classes for sophomore level biology majors should focus on biological application, rather than the math theory behind it. A biologist should need to know how and when to use a statistical test and how to interpret the data; the statistical equations behind it should be secondary. Such a course should be taught by a biologist who understands data analysis requirements, who is trained in R, Python, MATLAB, MEGA, PyMOL (Van Rossum and Drake, 1995; MatLab, 2010; PyMOL, 2010; Core R Team, 2017; Kumar et al., 2018) and can design multiple practical exercises for the course. Data on gene expression, heart rates, cholesterol levels, drug efficacy, biodiversity and community structure, evolutionary relationships, selection in a population, and mutations in a gene can be incorporated into class exercises thanks to easily accessible and free online databases and publications.

## Our Experience

### Computational Courses in a Biology Undergraduate Curriculum

To start filling the gaps in data analysis skills for future biologists, we offer a course on Bioinformatics (3-credits) for sophomores majoring in BS Biology at Kean University. The course is mandatory for the program option in Cell and Molecular Biology and is designed as a set of hands-on exercises on data analysis. Students use Excel and R statistical programming to work with biodiversity data, MEGA to study alignments and phylogenetics, and PyMOL for protein modeling. Introduction to R is taught in an online game form using DataCamp platform (datacamp.com). Since we found that most textbooks are too advanced for our sophomore undergraduates, we developed our own teaching activities. The activities are based on data available from NCBI, NEON, PDB databases, or our own research data (Shumskaya et al., 2019), and some even using the early nucleic acid and protein structure data on SARS-CoV-2 virus (Lorusso and Shumskaya, 2020; Shumskaya and Lorusso, 2020). The activities cited are published online as Open Education Resources to help promote our teaching philosophy surrounding bioinformatics.

For students interested in developing more skills in computational biology, we have developed a minor in Bioinformatics which includes an advanced course on biostatistics and a set of courses on basic computer programming that would count as free electives. This minor works for students majoring in computer sciences or informational technology as well; such students are required to have passed two semesters of general biology and genetics in addition to bioinformatics and statistics.

Additionally, we offer a Bioinformatics and Genomic Science track for our students pursuing a B.S. in Science and Technology in Molecular Biology. Students in this track complete courses in computer programming, statistical programming, and a bioinformatics elective by the end of their sophomore year. The responses from sophomores that complete our undergraduate bioinformatics course and related courses are overwhelmingly positive. In general, none of the students even considered computational biology as a field prior to participating in our bioinformatics course, being unfamiliar with this option. Course surveys reveal that most students become interested in the field when working with data, especially now when a lot of data on current COVID pandemic is available to practice (Johns Hopkins University Center for Systems Science and Engineering (CSSE), 2021) and appreciate learning new software that can help them succeed in multiple courses. A lack of student interest is a clear barrier that prevents students from pursuing higher-level courses that cover topics in bioinformatics (Williams et al., 2019). Our opinion is that a key component in getting students excited and interested in bioinformatics includes hands-on course exercises that cover real, timely, and relevant data (Shumskaya et al., 2019; Lorusso and Shumskaya, 2020; Shumskaya and Lorusso, 2020).

### Updating of Pre-Requisite Courses

Biology majors at Kean University are required to take a 3-credits course on statistics. This course traditionally has a lecture component with a heavy math approach; however, at Kean there is an option to add a 1 credit “Probabilistic Methods Lab” taught by a biologist. In this lab, students learn the R statistical programming language in the first half of the semester using the “Undergraduate Guide to R” tutorial (Martin, 2009). Programming activities are designed to reiterate key concepts such as data structures, functions, normalization of data, and graphs using the ggplot R package (Wickham, 2009), followed by the analysis of data such as drug efficacy and RNA expression levels. What separates this lab from a traditional computer programming courses is surrounding algorithms and equations are not detailed; rather, concepts and application of statistical tests using R are the focus. In this lab, students are “tool users” rather than “tool makers” (Pevsner, 2015). This enables students to analyze data and interpret results without the intimidation of complex equations. Because this course is given during their sophomore year, the exposure to basic computer programming has motivated some students to pursue more advanced computer programming courses during their junior or senior years. This approach helps us introduce bioinformatics into a lower level course, alleviating the “lack of student preparation” barrier (Williams et al., 2019).

### Computational Biology for Undergraduate Research

At Kean University, undergraduates can participate in undergraduate research courses [CUREs (Corwin et al., 2015; Rodenbusch et al., 2016; Shortlidge and Brownell, 2016)] such as Research-First-Initiative (RFI, freshmen) or Research Experience in Biology (REB, juniors). Such courses offer students an opportunity to join a faculty-led research project. The 2-3 credits courses are counted as a part of students’ 120-college credit program and are often used to jumpstart future independent research projects. Computational biology research projects are offered as part of CUREs. One RFI project focuses on understanding how mRNA localize within a developing cell. Specifically, freshmen are trained in quantifying real mRNA localization data from confocal images using custom MATLAB scripts from (Niepielko et al., 2018), and how to statistically analyze data and compare how mRNA localization changes in various genetic backgrounds. In two back-to-back semesters, students learn biological research such as single molecule *in situ* hybridization and confocal microscopy followed by computational analysis using MATLAB and R statistical programming. One REB course focuses on molecular biodiversity of dead wood decomposing fungi. Students work with Next Generation Sequencing to assess mycobiome gathered from environmental samples, and then employ a bioinformatics pipeline to analyze NGS data. This research course option finishes with students performing ordination and other statistical analyses to study microbial communities identified on dead wood. Research has shown that students engaging in research as undergraduates had the greatest benefit (Russell et al., 2007; Russell et al., 2017). By offering computational biology research and hands on training opportunities built into life science curricula, we believe that this addresses multiple educational barriers including lack of student interest, overly full curricula, and lack of student preparation. Together, we feel that our approach creates an environment that promotes bioinformatics while benefiting students (Levine, 2014) and faculty research projects.

### Summer Workshop for High School Students

We believe that bioinformatics and computational biology should be offered to students as early as possible. At Kean University, we offer a bioinformatics workshop for high school students that are interested in any STEM field. The 4 day remote workshop is offered during the summer months as part of Kean University’s Group Summer Scholars Research Program. The course is structured with a 2 h morning session and 2 h afternoon session which allows for students to receive a lecture on all the relevant background information in the morning and apply that knowledge by completing hands-on exercises in the afternoon. The hands-on activities cover RNA, DNA, and protein databases, BLAST searches, sequence analysis using MEGA, and protein structure analysis using PyMOL. Based on our experience, introducing bioinformatics to high school students has been overwhelmingly positive. Regardless of their diverse STEM interests, students are receptive to learning about the field and are proficient at completing all the workshop activities which include articulating key findings and developing hypotheses. Although Kean’s workshop is not part of a research study, we believe that offering general introduction to bioinformatics at a high school level will help relieve the “lack of student preparation” barrier identified in research studies (Williams et al., 2019). Furthermore, the feasibility and success of the workshop supports our opinion that early exposure to bioinformatics course material should be a strategy integrated into biology curricula and can be accomplished by including more background information into the course design rather than relying on mandatory pre-requisite courses, which should help alleviate overly full curricula issues.

## Discussion

It is no secret that many barriers exist that prevent exposing students to computational biology and bioinformatics, hence introduction of a special course on computational skills into undergraduate biology curricula is in dire need (Sayres et al., 2018; Williams et al., 2019). Our experience shows that the early introduction and a careful planning of computational biology courses has a positive influence on our diverse undergraduate student population. We summarized our steps on introducing computational biology in a biology curriculum in Table 1. Our goal is to promote and teach computational skills as early as possible so that students become comfortable with topics such as “How do I analyze data?” “When do I do a certain statistical test?” “What does the p-value actually mean?” In our opinion, biology students learning computer skills from other biologists helps students embrace quantitative biology without fear of overwhelming complex equations and computational algorithms.

**TABLE 1:** Introduction of computational skills in the biology curriculum.

Level	Biology major	Recommended intervention	Course content	Mandatory
Junior high school students	No	16 h summer workshop	Intro into RNA, DNA, and protein databases, BLAST searches, sequence analysis using MEGA, and protein analysis using PyMOL	No
Any college undergraduate level	Yes	Introductory research course on a specific topic, 1–2 credits	Hands-on analysis of real biological data acquired in lab or from publications	No
College freshmen/sophomores	Yes	1 semester of a lab course on statistics, 1 credit	Basics of R functions, ggplot graphing, interpreting graphs	Highly recommended
College sophomores	Yes	1 semester course on essentials of bioinformatics, 3 credits	Basics of working with biological data in Excel, R, MEGA, PyMOL. t-test, standard deviation and errors, ordination, BLAST, multiple sequence alignment, phylogeny, protein modeling, selection. Online databases NCBI, PDB etc.	Yes
College juniors, seniors	Yes	1 semester course on biostatistics, 3 credits	Advanced data analysis in Excel and R	Yes for minors in bioinformatics; major elective for others

From our experience, providing an early opportunity for students to get involved with computational biology spikes their interest to continue to more advanced independent research projects, especially if they participate in CUREs. In a broader sense, such training would have a huge impact on our society. As documented with COVID-19 analyses we discussed above, scientific data can be misrepresented very easily, leading towards rapid spread of misinformation and poor policy choices. The more specialists that receive training in data analysis and data interpretation, the better, regardless of their specialized background. In the future, perhaps general education courses on data analysis and data interpretation can be designed and made a requirement for all student majors.
